# Supplementary material for: Diabetes knowledge in nursing homes and home-based care services: a validation study of the Michigan Diabetes Knowledge Test adapted for use among nursing personnel
Source: BMC Nurs. 2016 Jun 29;15:40. doi: 10.1186/s12912-016-0159-1 (PMC4928289; doi:10.1186/s12912-016-0159-1)
Supplement: Additional file 1: — The adapted Michigan Diabetes Knowledge Test. (DOC 519 kb) [file 12912_2016_159_MOESM1_ESM.doc]

# **Diabetes knowledge in nursing homes and home-based care services: a validation study of the Michigan Diabetes Knowledge Test adapted for use among nursing personnel**

Anne Haugstvedt^a^, Morten Aarflot^b^, Jannicke Igland^a,c^, Tilla Landbakk^d^ and Marit Graue^a^

^a^Faculty of Health and Social Sciences, Centre for Evidence-Based Practice, Bergen University College, ^b^Department of Community Medicine, University of Tromsø, ^c^Department of Global Public Health and Primary Care, University of Bergen, Bergen, ^d^Innlandet Hospital Trust, Gjøvik, Norway

Corresponding author:

Anne Haugstvedt

Faculty of Health and Social Sciences, Bergen University College

Post Box 7030, N-5020 Bergen, Norway

Phone: + 47 55 58 78 88, Mobile + 47 47 82 92 20

E-mail: [ahau@hib.no](mailto:ahau@hib.no)

Co-authors’ e-mail addresses:

Morten Aarflot: [morten.aarflot@uit.no](mailto:morten.aarflot@uit.no)

Jannicke Igland: [jannicke.igland@uib.no](mailto:jannicke.igland@uib.no)

Tilla Landbakk: [Tilla.L.Landbakk@sykehuset-innlandet.no](mailto:Tilla.L.Landbakk@sykehuset-innlandet.no)

Marit Graue: [mgr@hib.no](mailto:mgr@hib.no)

Word count: 3566

**Background** Providing high-quality diabetes care in nursing homes and home-based care facilities requires suitable instruments to evaluate the level of diabetes knowledge among the health-care providers. Thus, the aim of this study was to examine the psychometric properties of the Michigan Diabetes Knowledge Test adapted for use among nursing personnel.

**Methods** The study included 127 nursing personnel (32 registered nurses, 69 nursing aides and 26 nursing assistants) at three nursing homes and one home-based care facility in Norway. We examined the reliability and content and construct validity of the Michigan Diabetes Knowledge Test.

**Results** The items in both the general diabetes subscale and the insulin-use subscale were considered relevant and appropriate. The instrument showed satisfactory properties for distinguishing between groups. Item response theory-based measurements and item information curves indicate maximum information at average or lower knowledge scores. Internal consistency and the item-total correlations were quite weak, indicating that the Michigan Diabetes Knowledge Test measures a set of items related to various relevant knowledge topics but not necessarily related to each other.

**Conclusions** The Michigan Diabetes Knowledge Test measures a broad range of topics relevant to diabetes care. It is an appropriate instrument for identifying individual and distinct needs for diabetes education among nursing personnel. The knowledge gaps identified by the Michigan Diabetes Knowledge Test could also provide useful input for the content of educational activities. However, some revision of the test should be considered.

Keywords: diabetes – elderly – nursing personnel – Michigan Diabetes Knowledge Test

**Background**

The rising worldwide prevalence of diabetes and the increasing older population in many societies mean that the number of older people with diabetes is expected to increase in the years to come [1,2]. Older people with diabetes may have more difficulty in managing the self-care required by diabetes because of escalating physical and cognitive problems, thus increasing the need for home-based care services and nursing home residence [1,3–5]. Further, adequate and appropriate diabetes knowledge among the nursing personnel providing care for older people with diabetes is required to meet the complex daily care needs among this group of older people. However, insufficient diabetes knowledge among nursing personnel and a consistent lack of adherence to evidence-based recommendations and guidelines have previously been identified in community care [6–9]. Ensuring adequate diabetes knowledge to provide high-quality diabetes care in community care settings requires suitable instruments to evaluate the level of diabetes knowledge among nursing personnel. A published review-article from 2013 on existing instruments to assess diabetes knowledge among nurses, advocates updated and sound instruments to assess diabetes knowledge among nurses [10]. This study therefore aimed to examine the psychometric properties of an adapted version of the Michigan Diabetes Knowledge Test (MDKT) for use among nursing personnel (registered nurses, nursing aides and nursing assistants) regarding its reliability (including internal consistency and item-total correlations) and content and construct validity (including item response theory analysis and the ability to distinguish between groups). We hypothesized the following:

1. Registered nurses, nursing aides and nursing assistants who report experiencing their diabetes knowledge as sufficient, will obtain higher scores on both the MDKT general diabetes subscale and the insulin-use subscale than those who report experiencing their diabetes knowledge as insufficient in relation to their work tasks.
2. Registered nurses will obtain higher scores than nursing aides and nursing assistants on both MDKT subscales.
3. Those who report being delegated to administer insulin will report higher scores on both MDKT subscales than those who have not been delegated.

**Methods**

*Design and participants*

We invited six experts to participate in translating the MDKT into Norwegian, including adaptation for use among nursing personnel. In addition, we pilot tested the Norwegian version among eight nursing personnel to assess the face validity.

In the main cross-sectional study, 127 nursing personnel participated: 37 (29%) registered nurses, 69 (54%) nursing aides and 21 (17%) nursing assistants (no formal health education). Table 1 shows the demographic characteristics of the participants recruited from three nursing homes and one home-based care facility in Norway.

*Questionnaire*

In addition to the MDKT, the study questionnaire included demographic data, items about the participants’ employment status (“full-time work”, “temporary employment status” and “hourly based”), work experience in a nursing home or home-based care ( “<1 year”, “1–5 years” and “>5 years”), delegation to administer insulin (yes/no) and a single item about the participants’ perception of their diabetes knowledge being sufficient or not in relation to given work tasks (yes/no).

The original MDKT included 23 items divided into two subscales: the general diabetes subscale with 14 items (items 1–14) (e.g. “Which should not be used to treat low blood glucose?”) and the insulin-use subscale with nine items (items 15–23) (e.g. “If you are sick with the flu, which of the following changes should you make?”) ([http://www.med.umich.edu/mdrtc/profs/survey.html#dkt](http://www.med.umich.edu/mdrtc/profs/survey.html#dkt)). Each item has three or four answer categories; one is correct. Before analysis, we recoded the answer categories into two categories, correct (= 1) or wrong (= 0), and calculated a sum score for each participant as the percentage of correct answers. The MDKT, which was developed for use among adults with type 1 or type 2 diabetes, has previously shown appropriate reliability and validity [11]. The test was translated into Norwegian for this study using the academic translation procedure recommended by WHO ([www.who.int/substance_abuse/research_tools/translation/en](http://www.who.int/substance_abuse/research_tools/translation/en)). To adapt the MDKT for use among nursing personnel, pronouns were adjusted (e.g. “If you are sick with the flu …” was changed to “If a person with diabetes is sick with the flu …”). The originator of the MDKT at the Michigan Diabetes Research and Training Center approved both the back-translation of the MDKT and the adaptation for use among nursing personnel. The MDKT was adjusted previously in a study among mothers of children with diabetes [12]. Appendix 1 shows the Norwegian version of the adapted MDKT.

*Ethical considerations*

Ethics committee approval was not required for this study, but the Data Protection Official for Research approved the study, which was performed according to the guidelines for research ethics prepared by the Norwegian National Committees for Research Ethics. The participants received both oral and written information about the study, and the completed questionnaire was considered as informed consent. The questionnaires were completed at work, and the participants were encouraged to not collaborate with colleagues.

*Analysis*

In all analyses, we analysed the MDKT general diabetes subscale and the MDKT insulin-use subscale separately. We report descriptive statistics as counts, proportions, means and standard deviation (SD) to characterize the sample and their scores on the MDKT subscales and measure the internal consistency reliability of the subscale scores by using standardized Cronbach’s alpha, which is equivalent to the Kuder-Richardson Formula 20 (KR-20) for binary items [13,14]. To identify items that are not consistent with the rest of the instrument, we calculated corrected item-total correlations by correlating each single item score with the total score obtained by summing all the other items in the subscale, not including the item in question.

We used item response theory–based measurements (one-parameter Rasch model) to analyse response patterns and how individual items perform within the current MDKT subscales. The Rasch model assumes that the probability of a person answering an item correctly depends on the respondent’s underlying trait level and the item difficulty [15,16]. For the MDKT, the underlying trait is diabetes knowledge, referring to a broad range of topics relevant to diabetes management and treatment. The item difficulty estimated from the Rasch model quantifies the difficulty parameter for each individual item, with low values reflecting easy items. We summarized the performance of each item by an item characteristic curve and an item information curve (Fig. 1). The item characteristic curve for an item displays the estimated probability that a respondent answers correctly as a function of the underlying latent knowledge level. The underlying knowledge is displayed as a standardized knowledge score, where a score equal to zero is the average knowledge level, the value –1 reflects a score one standard deviation below the average and +1 reflects a score one standard deviation above the average. A curve that increases from left to right indicates that a respondent with a high level of knowledge has a higher probability of obtaining a correct answer than respondents with a lower level of knowledge; a flat curve indicates that a respondent with a high level of knowledge has the same probability of obtaining a correct answer as a respondent with a low level of knowledge. This would suggest that the item should be considered to be eliminated from the test, since it cannot be used to differentiate between respondents with high and low diabetes knowledge. The item information curve for an item reflects the level of information across different levels of knowledge. The highest point on the curve represents the knowledge level at which the item provides the most information. High information at low knowledge levels indicates that the specific item is best suited to differentiate between respondents with lower knowledge. We obtained the total test information for each subscale as the sum of the information curves for each item in the subscale. We assessed the adequacy of the one-parameter Rasch models using a goodness-of-fit test comparing the observed response patterns with the expected patterns according to the model and by the Akaike information criterion, for which lower values indicate better fit.

We used independent sample *t*-tests to test our first hypothesis regarding differences in MDKT subscale scores between those who experience their own diabetes knowledge as sufficient versus insufficient. We used analysis of variance (ANOVA) to test the second hypothesis regarding the subscales’ ability to distinguish between the three professions. We also analysed this by analysis of covariance (ANCOVA) and included the duration of the participants’ work experience within the nursing home or home-based care setting as a covariate. We analysed the third hypothesis regarding the subscales’ ability to distinguish between those who administered insulin and those who did not by independent sample *t*-test.

We defined statistical significance as *P* < 0.05 and used SPSS version 22 (IBM SPSS, Armonk, NY, USA). We performed analysis and item characteristic curves in the program R (R Foundation for Statistical Computing, Vienna, Austria) using the ltm-package [17].

**Results**

*Reliability*

The Norwegian version of the MDKT showed quite weak internal consistency for the general diabetes subscale (standardized Cronbach’s alpha: 0.57) and for the insulin-use subscale (standardized Cronbach’s alpha: 0.42), implying that considering knowledge as a single underlying construct when the questions included in a scale refer to various topics (as the MDKT does) might be problematic. The item-total correlations (Table 2) were >0.2 for items 3, 5, 6 and 10–14 in the general diabetes subscale and >0.2 for items 20–22 in the insulin-use subscale.

*Content validity*

The expert group experienced the items in the general diabetes subscale as relevant and suitable independent of diabetes treatment regimen. The knowledge required in the insulin-use subscale items was considered relevant for nursing personnel working with insulin users. The participants in the pilot study experienced the items in the MDKT as being relevant and appropriate. They presented some minor remarks on the wording, and we made some minor changes. The authors of the MDKT confirmed a back-translated version.

*Construct validity*

The estimated item difficulties from the 1-parameter Rasch model (Table 2) indicated that item 19 was the easiest item, with a difficulty parameter of –6.55 and predicted probability of obtaining a correct answer of 99%. This was also reflected in the item characteristic curve for item 19 (Fig. 1), which showed that the probability of obtaining a correct answer was close to 1 regardless of knowledge level. The most difficult item was item 4, with a difficulty parameter of 2.35 and a predicted probability of obtaining a correct answer of only 16%. Apart from item 19 in the insulin-use subscale and item 6 in the general diabetes subscale, the item characteristic curves for the remaining items showed a clear increase in the probability of obtaining a correct answer from low knowledge levels to high knowledge levels (Fig. 1), indicating that the probability of obtaining a correct answer is associated with the total knowledge level of the respondents. For instance, for item 1, the probability of obtaining a correct answer was 20% for respondents with knowledge scores 4 standard deviations below the average, 80% for respondents with average knowledge scores and close to 100% for respondents with knowledge scores 4 standard deviations above the average. Respondents with less-than-average knowledge scores had a less than 20% probability of obtaining a correct answer for the item characteristic curve for the most difficult item (item 4) and increasing probability for respondents with higher-than-average knowledge scores. The item information curves generally had maximum information at average or lower knowledge scores. The total test information curves (Fig. 2) showed maximum information at about 2 standard deviations below the average knowledge level. The goodness-of fit-test, as determined by 200 bootstraps of Pearson’s chi-square, indicated lack of fit in the general diabetes subscale (*P* = 0.02) and no lack of fit in the insulin-use subscale (*P* = 0.06). After we excluded item 6 in the general diabetes subscale and item 19 in the insulin-use subscale from the Rasch models, the *P*-values for the goodness-of-fit tests increased to 0.18 and 0.7, respectively. For the general diabetes scale, removing item 6 also reduced the Akaike information criterion from 1694.0 to 1640.0, and removing item 19 from the insulin-use subscale improved the Akaike information criterion from 821.6 to 800.1.

Related to the participants’ experience of their own diabetes knowledge as sufficient or not related to their given work tasks, 50 (41%) participants reported their diabetes knowledge as insufficient. As hypothesized, we identified statistically significant differences in the general diabetes subscale scores between those who experienced their own diabetes knowledge as sufficient and those who did not (*P* < 0.01) (Table 3).

Table 3 indicates statistically significant differences between nurses, nursing aides and nursing assistants in both general diabetes subscale scores and insulin-use subscale scores, in accordance with our second hypothesis. The registered nurses had the highest percentage of correct answers and the nursing assistants the lowest percentage of correct answers in both subscales. The differences between the groups remained significant also after adjustment for the duration of work experience. Regarding our third hypothesis, we also identified significant differences between those who had been delegated to administer insulin and those who had not for the general diabetes subscale (*P* < 0.01) (Table 3). Those who had been delegated to administer insulin also scored higher on the insulin-use subscale than those who had not, but the difference was not statistically significant.

**Discussion**

The results indicate that both the MDKT general subscale and the insulin-use subscale adapted for nursing personnel can be recommended for further use, but some modification of the subscales should be considered based on the results from the measurements performed in this study.

Both the expert group and the participating nursing personnel in this study experienced the knowledge required in the MDKT as relevant. The topics included in the general diabetes subscale were considered to be relevant for all types of diabetes and treatment regimens related to both type 1 and type 2 diabetes. Further, the items in the insulin-use subscale were considered to be relevant to insulin treatment. The subscale requires knowledge that nursing personnel should have when working with people taking insulin. These findings are in contrast with previous research that claimed that MDKT is an outdated instrument [18]. A potential weakness, however, is that the MDKT does not include questions related to specific new treatment regimens such as insulin pump treatment or various types of glucose-lowering medications for type 2 diabetes. A suggestion could be to develop additional subscales focusing on insulin-pump use, newer types of insulin, various types of glucose-lowering medications for type 2 diabetes and maybe also questions related to blood pressure and lipid-lowering actions. However, in accordance with Benetos et al. [19], we emphasize the need for essential and basic knowledge related to high and low blood glucose among nursing personnel in nursing homes and home-based care as being among the most important knowledge to secure satisfactory diabetes management and subsequently the best possible daily life for older people with diabetes. A study among 100 nursing home residents with diabetes [20] indicated and highlighted unsatisfactory recognition of especially low blood glucose and its negative consequences for the residents’ quality of life. Accordingly, Garcia & Brown [8] also highlight in their review of the literature the high prevalence of adverse events among nursing home residents with diabetes such as hypoglycaemia, hospitalization, skin ulcers, infections and amputations, but they indicate that the literature unfortunately does not associate these with diabetes management characteristics.

Regarding the psychometric properties of the MDKT, we identified relatively weak internal consistency and item-total correlations for both subscales. A Cronbach’s alpha of at least 0.70 is generally recommended in developing psychometric instruments [16]. One could, however, question whether Cronbach’s alpha and item-total correlations are appropriate measures for this kind of knowledge test. The use of Cronbach’s alpha as a measure of internal consistency has been criticized [21]. It has also been argued that a high Cronbach’s alpha would not be expected for index-type questionnaires in which the items themselves define the construct and can include unrelated items, in contrast to questionnaires in which the items measure a single underlying construct such as anxiety or depression [22]. When an index is defined to measure a construct such as knowledge, it might be of interest to select items that are related to the construct of interest but not necessarily to each other. The general diabetes subscale is meant to capture a broad range of topics relevant to diabetes (e.g. dietary concerns and the effect of nutrition on blood glucose control, how to measure blood glucose regulation, how to treat hypoglycaemia, physical activity and long-term complications). The insulin-use subscale includes items on essential aspects critical for administering insulin treatment (e.g. how to treat diabetes when people have infections, signs of ketoacidosis, blood glucose–lowering effect of insulin, reasons for hyperglycaemic events and what to do when insulin doses are forgotten). Nursing personnel may have knowledge about some of the required topics without having knowledge about other topics. Thus, in our opinion, considering knowledge as a single underlying construct of the MDKT subscales is difficult. It is more reasonable to classify the MDKT as an index-type questionnaire according to the definition used by Streiner [22]. Lack of a single underlying construct is probably the main reason for the weak consistencies and correlations identified for the MDKT subscales in this study.

Related to the construct of the MDKT subscales, the total test information curves showed maximum information at about 2 standard deviations below the average knowledge level, indicating that the MDKT is a rather easy test that is best suited to distinguish between respondents on the lower end of the knowledge scale. This may indicate that the MDKT could be an appropriate instrument for use in community care to identify individual and distinct needs for diabetes education among nursing personnel. Knowledge gaps identified by the MDKT could also provide useful input for planning and implementing educational activities and training courses. Although publications on educational activities for nursing home employees are scarce, such activities have been shown to improve the quality of diabetes care in nursing homes [23].

In accordance with the MDKT’s property of distinguishing between respondents on the lower end of the knowledge scale, it might be more important to achieve satisfactory quality of diabetes care by identifying the nursing personnel lacking basic diabetes knowledge rather than instruments that are more sophisticated at differentiating between those at the upper level of the scale. Nevertheless, we recommend some modification of the MDKT. Based on our results, we suggest removing item 6 (general diabetes subscale) and item 19 (insulin-use subscale). These items seem to be too easy, since almost all participants answer them correctly. The model-fit (the goodness-of-fit test) also improved when these items were removed. Further, items 7 and 9 (general diabetes subscale) seem to be quite easy and do not contribute significantly to differentiating between groups of respondents.

The original MDKT subscales were developed for use among adults with diabetes. It is reasonable to claim that nursing personnel working with people with diabetes should have at least the same theoretical diabetes knowledge as the patients and preferably a higher level of knowledge. Nevertheless, we could not compare our results with other studies since we did not find any others that have validated the psychometric properties of the MKDT among nursing personnel. In nursing homes and home-based care, the nursing personnel must often take over the patients’ self-care. Basic knowledge is sufficient to do this satisfactorily. The scores on the MDKT subscales in this study indicate a knowledge gap related to several important topics. For example, the registered nurses, nursing aides and nursing assistants lack knowledge about the signs of ketoacidosis. The registered nurses scored correctly on 76% of the questions in the general diabetes subscale and 79% of the questions in the insulin-use subscale. This indicates potential for improvement. As hypothesized, registered nurses scored higher than nursing aides and nursing assistants on both subscales, which could contribute to a discussion of the distribution of the professionals employed in nursing homes and home-based care facilities. Further, discussing the diabetes knowledge required in nursing homes and home-based care is strongly needed, since this study indicated that 41% of the respondents experienced their own diabetes knowledge as insufficient in relation to their given work tasks. For the general diabetes subscale, own experience of diabetes knowledge was significantly correlated with the subscale score. In this study, the insulin-use subscale did not show the same properties of differentiating between groups as the general diabetes subscale.

This study has some limitations. The sample was relatively small and limited the possibilities for further subgroup analyses to explore the reliability and validity of the MDKT in different groups of professionals, separately. Further, we did not test whether the MDKT subscales are sensitive and appropriate scales to measure the effect of educational interventions. As commented also by the developers of the scale [11], the test may not be sensitive for all aspects and components of diabetes education and care, although the test includes several important topics. Further research and further discussions are needed related to the topics and items included in the subscales, their psychometric properties and how to use the MDKT subscales appropriately.

**Conclusions**

The MDKT, including the general diabetes subscale and the insulin-use subscale, is perceived as a relevant and appropriate instrument for measuring diabetes knowledge among nursing personnel in nursing home and home-based care. The MDKT is an appropriate instrument for identifying individual and distinct needs for diabetes education. Further, the design and implementation of educational activities and training courses could be adapted to target the specific needs of knowledge revealed by the MDKT. However, some revision of the MDKT should be considered.

**Abbreviations** — MDKT, Michigan Diabetes Knowledge Test

**Ethics approval and consent to participate**

The Norwegian Data Protection Official for Research approved the study. Ethics committee approval was not required. The participants gave written consent and approved publication of results.

**Availability of data and materials**

Due to ethical and legal restrictions related to confidentiality, the data is not deposited online as the study participants have not explicitly been informed about, nor approved data sharing when informed consent were gathered.

**Competing interests**

No competing interests to declare.

**Funding**

Bergen University College, Bergen Municipality and the Norwegian Research Council funded the study.

**Author contributions**

AH designed the study, collected the data, analysed the data and drafted the manuscript; MA and JI assisted in the statistical analysis and contributed to the final manuscript; TL and MG contributed to designing the study, collecting the data and drafting the manuscript. All authors read and approved the final manuscript.

**Acknowledgements**

We thank all the nursing personnel who participated. We especially thank nurses Linda Heimvik and Ann Kristin Akselberg, who carried out important parts of the data collection.

# References

1. International Diabetes Federation (IDF). *IDF Diabetes Atlas*. Sixth edition. 2014. Available at <http://www.idf.org/diabetesatlas>; last accessed 11 April 2016.
2. Kirkman MS, Briscoe VJ, Clark N, Florez H, Haas LB *et al*. Diabetes in older adults. Consensus report. *Diabetes Care* 2012; **35**: 2650–2664.
3. Shaw JE, Sicree RA, Zimmet PZ. Global estimates of the prevalence of diabetes for 2010 and 2030. *Diabetes Res Clin Pract* 2010; **87**: 4–14.
4. Bourdel-Marchasson I, Berrut G. Caring the elderly diabetic patient with respect to concepts of successful aging and frailty. *Diabetes Metab* 2005; 31 Spec No 2: 5S13–5S19.
5. Sinclair A, Dunning T, Colagiuri S, International Diabetes Federation (IDF) Working Group. *IDF Global Guideline for* [*Managing Older People with Type 2 Diabetes*](http://www.idf.org/guidelines/managing-older-people-type-2-diabetes). 2012. Available from <http://www.idf.org/sites/default/files/IDF-Guideline-for-Type-2-Diabetes.pdf>; last accessed 11 April 2016.
6. Ödegård S, Andersson DK. Knowledge of diabetes among personnel in home-based care: how does it relate to medical mishaps? *J Nurs Manage* 2001; 9: 107–114.
7. [Berlowitz DR](http://www.ncbi.nlm.nih.gov/pubmed/?term=Berlowitz DR%5BAuthor%5D&cauthor=true&cauthor_uid=11816849), [Young GJ](http://www.ncbi.nlm.nih.gov/pubmed/?term=Young GJ%5BAuthor%5D&cauthor=true&cauthor_uid=11816849), [Hickey EC](http://www.ncbi.nlm.nih.gov/pubmed/?term=Hickey EC%5BAuthor%5D&cauthor=true&cauthor_uid=11816849), [Joseph J](http://www.ncbi.nlm.nih.gov/pubmed/?term=Joseph J%5BAuthor%5D&cauthor=true&cauthor_uid=11816849), [Anderson JJ](http://www.ncbi.nlm.nih.gov/pubmed/?term=Anderson JJ%5BAuthor%5D&cauthor=true&cauthor_uid=11816849), [Ash AS](http://www.ncbi.nlm.nih.gov/pubmed/?term=Ash AS%5BAuthor%5D&cauthor=true&cauthor_uid=11816849) [*et al*](http://www.ncbi.nlm.nih.gov/pubmed/?term=Moskowitz MA%5BAuthor%5D&cauthor=true&cauthor_uid=11816849). Clinical practice guidelines in the nursing home. [*Am J Med Quality*](http://www.ncbi.nlm.nih.gov/pubmed/11816849) 2001; **16**:189–195.
8. Garcia TJ, Brown SA. Diabetes management in the nursing home: a systematic review of the literature. *Diabetes Educ* 2011; 37:167–187.
9. Vajen BM, Holt R, Marx T, Schwartz FL, Shubrook Jr JH. How well are we managing diabetes in long-term care? *J Fam Pract* 2012; **61**: 467–472.
10. Francisco MA. Instruments that measure nurses’ knowledge about diabetes: an integrative review. *J Nurs Measure* 2013; **21**: 137–152.
11. Fitzgerald JT, Funnell MM, Hess GE, Barr PA, Anderson RM, Hiss RG *et al*. The reliability and validity of a brief diabetes knowledge test. *Diabetes Care* 1997; **21**: 706–711.
12. Tahirovic H, Toromanovic A. Glycemic control in diabetes children: role of mother’s knowledge and socioeconomic status. *Eur J Pediatrics* 2010; **169**: 961–964.
13. Streiner DL. Starting at the beginning: An introduction to coefficient alpha and internal consistency. *J Pers Assess* 2003; **80**: 99–103.
14. Field A. *Discovering statistics using SPSS (and sex, drugs and rock’n’roll)*. London: SAGE Publications, 2012.
15. Furr RM, Bacharach VR. *Psychometrics: an introduction*. Second edition. Los Angeles: SAGE Publications, 2014.
16. DeVellis RF. *Scale development: theory and applications.* Third edition. Los Angeles: SAGE Publications, 2012.
17. Rizopoulos, D. An R package for latent variable modelling and item response theory analyses.*J Stat Software* 2006; **17**(5): 1–25. Available from [http://www.jstatsoft.org/v17/i05;](http://www.jstatsoft.org/v17/i05/) last accessed 11 April 2016.
18. Quandt SA, Ip EH, Kirk JK, Saldana S, Chen S-H, Nguyen H *et al*. Assessment of a short diabetes knowledge instrument for older and minority adults. *Diabetes Educ* 2014; **40**: 68–76.
19. Benetos A, Novella JL, Guerci B, Blickle JF, Boivin JM, Cuny P *et al*. Pragmatic diabetes management in nursing homes: individual care plan. *J Am Med Dir Assoc* 2013; **14**: 791–800.
20. Andreassen LM, Sandberg S, Kristensen GBB, Solvik UO, Kjome RLS. Nursing home patients with diabetes: prevalence, drug treatment and glycemic control. *Diabetes Res Clin Pract* 2014; **105**:102–109.
21. Sijtsma K. On the use, the misuse, and the very limited usefulness of Cronbach’s alpha. *Psychometrika* 2009; **74**: 107–120.
22. Streiner DL. Being inconsistent about consistency: when coefficient alpha does and doesn't matter. *J Pers Assess* 2003; **80**: 217–222.
23. Hager KK, Loprinzi P, Stone D. Implementing diabetes care guidelines in long-term care. *J Am Med Dir Assoc* 2013; **14**: 851e7–851e15.

Table 1 Demographic characteristics for 127 nursing personnel in three Norwegian nursing homes and one home-based care facility

| **Characteristics** | ***n* (%)** |
| --- | --- |
| **Profession (*n* = 127)** |  |
| Registered nurses | 37 (29) |
| Nursing aides | 69 (54) |
| Nursing assistants | 21 (17) |
| **Employment (*n* = 125)** |  |
| Permanent | 111 (89) |
| Temporary | 14 (11) |
|  |  |
| **Work experience (*n*= 127)** |  |
| <1 year | 6 (5) |
| 1–5 years | 29 (23) |
| <5 years | 92 (72) |

Table 2 Item-total correlations for the MDKT subscales among 127 nursing personnel in nursing homes and home-based care

| **Component^a^** | **Percentage correct** | **Item-total correlation** | **Item difficulty (Rasch analysis)** |
| --- | --- | --- | --- |
| **General test (items 1–14)** |  |  |  |
| *n* = 127 |  | |  |
| 1 | 79.5 | 0.14 | –2.23 |
| 2 | 48.0 | 0.07 | –0.10 |
| 3 | 55.9 | 0.30 | –0.57 |
| 4 | 16.5 | 0.10 | 2.35 |
| 5 | 52.0 | 0.42 | –0.50 |
| 6 | 91.3 | 0.38 | –4.06 |
| 7 | 85.0 | 0.08 | –3.42 |
| 8 | 55.1 | 0.15 | –0.40 |
| 9 | 88.2 | 0.17 | –3.42 |
| 10 | 81.1 | 0.21 | –2.60 |
| 11 | 80.3 | 0.29 | –2.45 |
| 12 | 78.7 | 0.33 | –2.28 |
| 13 | 64.6 | 0.27 | –1.25 |
| 14 | 79.5 | 0.20 | –2.50 |
| Insulin use (items 15–23) |  |  |  |
| *n* = 127 |  | |  |
| 15 | 40.9 | 0.00 | 0.14 |
| 16 | 84.3 | 0.04 | –3.69 |
| 17 | 52.8 | 0.05 | –0.44 |
| 18 | 71.7 | 0.14 | –1.90 |
| 19 | 95.3 | 0.08 | –6.55 |
| 20 | 81.1 | 0.46 | –3.04 |
| 21 | 84.3 | 0.27 | –3.22 |
| 22 | 81.1 | 0.33 | –3.05 |
| 23 | 53.5 | 0.18 | –0.39 |

^a^The discrimination coefficients were 0.66 for the diabetes general test and 0.71 for the insulin-use subscale.

Table 3 Differences in the mean percentage of correct answers on the MDKT between groups of professions, groups related to experienced own diabetes knowledge and groups related to insulin administration (*n* = 127^a^) in nursing homes and home-based care

| **MDKT** |  | **General diabetes**  **subscale**  (items 1–14) | **Insulin-use**  **subscale**  (items 15–23) |
| --- | --- | --- | --- |
|  | *n* | Mean % correct answers (SD) | Mean % correct answers (SD) |
| Total | 127 | 69.4 (15.1) | 73.4 (17.6) |
| Profession |  |  |  |
| Registered nurses | 36 | 76.4 (14.7)^d^ | 79.0 (14.7) |
| Nursing aides | 67 | 66.9 (14.2) | 73.3 (16.5) |
| Nursing assistants | 21 | 65.3 (15.2) | 64.0 (21.6)^d^ |
| *P*-value for differences between the groups^b^ |  | <0.01^d^ | <0.01^d^ |
| Experience of own diabetes knowledge as sufficient |  |  |  |
| Yes | 72 | 72.5 (13.3) | 74.8 (18.6) |
| No | 50 | 65.1 (16.1) | 71.1 (16.0) |
| *P*-value for differences between the answer categories^c^ |  | <0.01^d^ | 0.25 |
| Delegation to administer insulin |  |  |  |
| Yes | 82 | 73.6 (14.4) | 74.9 (17.4) |
| No | 40 | 60.0 (12.4) | 70.6 (18.3) |
| *P*-value for differences between the answer categories^c^ |  | <0.01 | 0.22 |

^a^One nursing aide and one registered nurse did not answer the questions on the general diabetes subscale, and two nursing aides and one nurse did not answer the questions on the insulin-use subscale.

^b^*P*-value for differences between groups tested by ANOVA.

^c^*P*-value for differences between groups tested by independent sample *t*-test.

^d^*P* < 0.05.

**Additional files**

*Additional files 1 and 2:*

Figure 1 The item characteristic curves (ICC) (solid line) and item information curves (IIC) (dashed line) for diabetes knowledge items related to the MDKT adapted for nursing personnel

*The figure is submitted in two separate files (one for the general diabetes subscale and one for the insulin-use subscale) included one curve for each item. The name of these additional files are:*

*Figure1a_ITRcurves_MDKTsubscale1.eps*

*Figure1b_ITRcurves_MDKTsubscale2.eps*

*Additional file 3:*

Figure 2 Test information curve for the general diabetes test and the insulin-use test in MDKT

*The name of the file is:*

*Figure2_TestInformationCurve_MDKT.eps*

*Additional file 4:*

Appendix 1

*The adapted Michigan Diabetes Knowledge Test is submitted in a separate file named:*

*BacktranslatedVersionOfNorwegianMDKT_forHP_20.09.15*


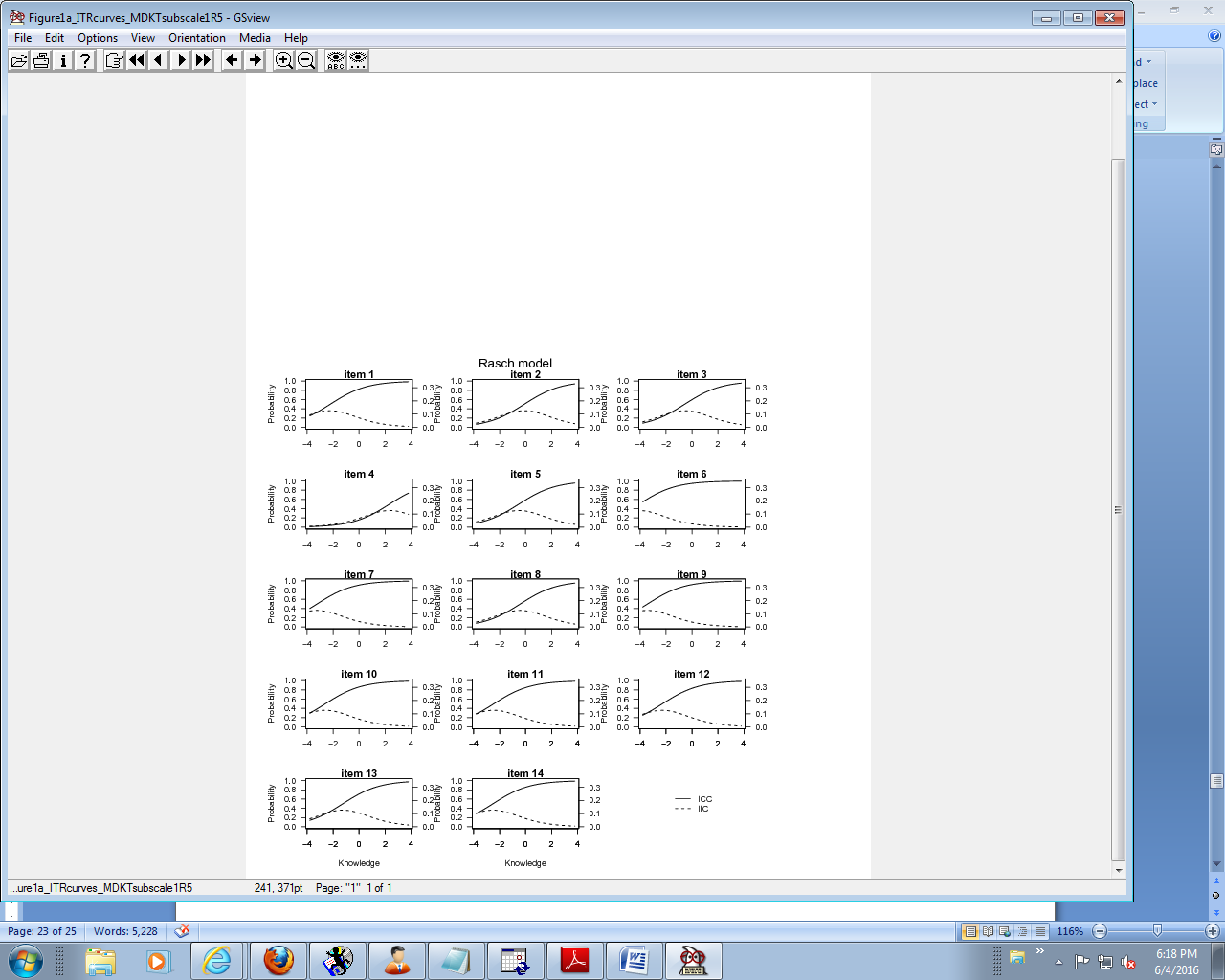


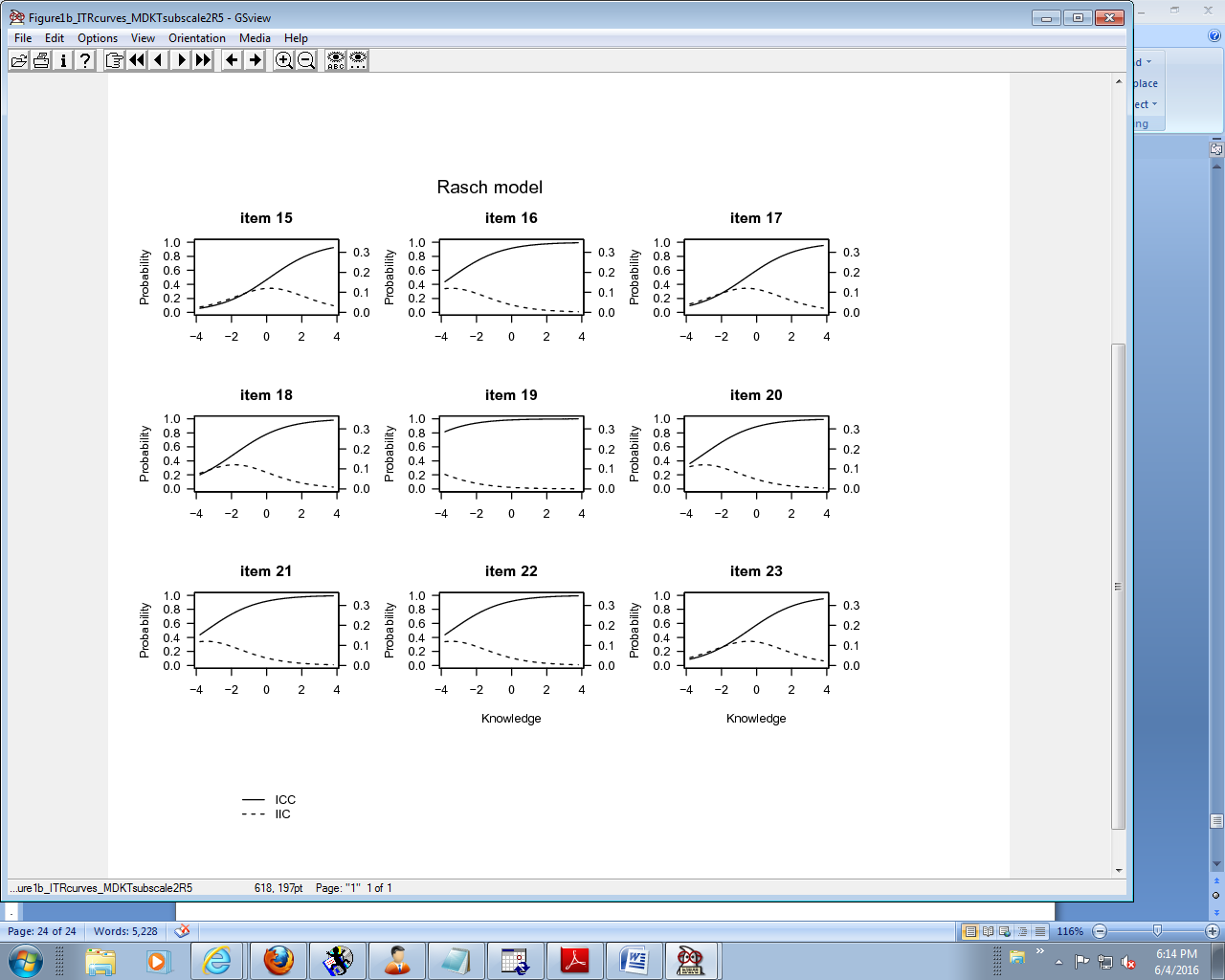


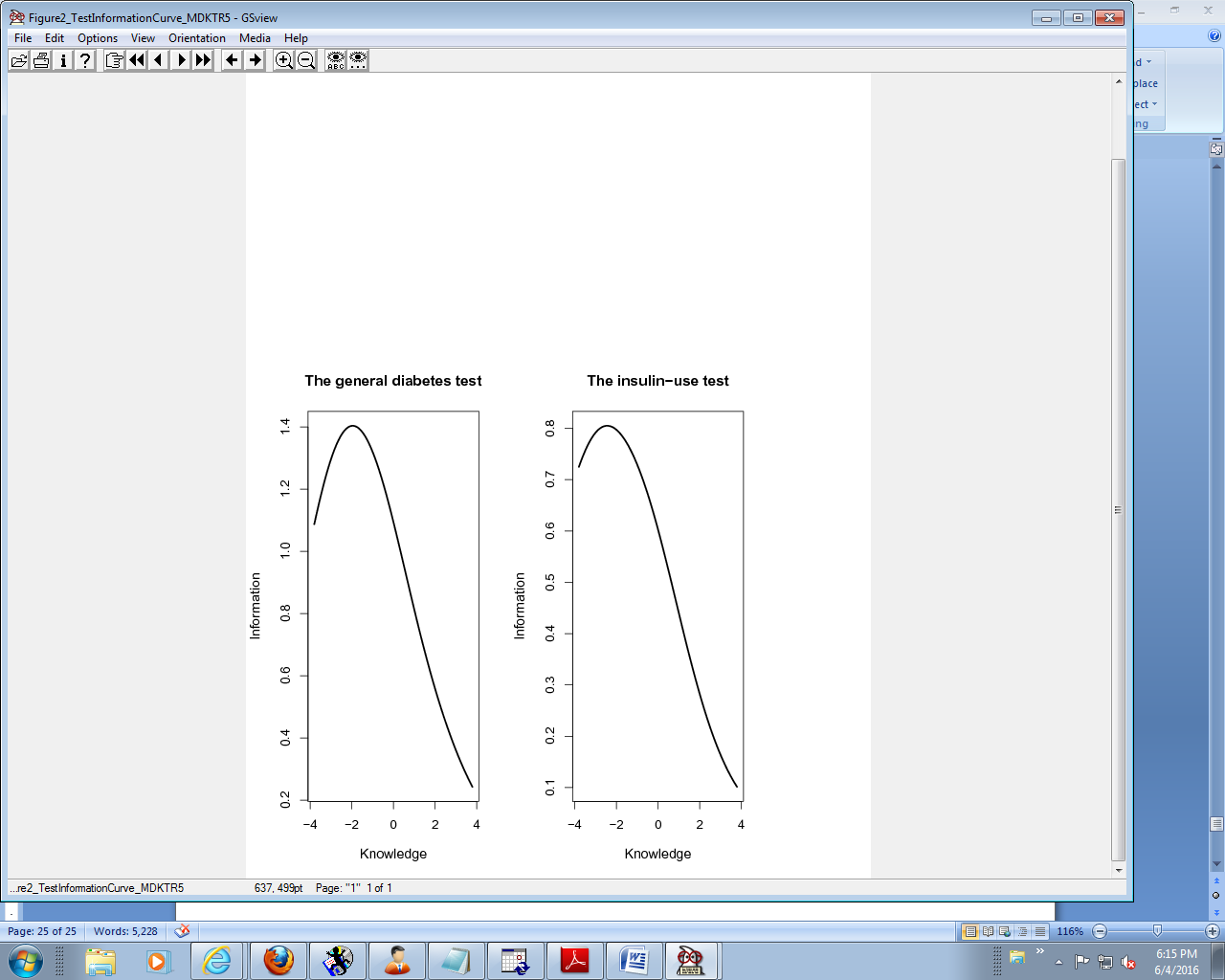


**Michigan Diabetes Knowledge Test (Michigan DKT)**

**-for nursing personnel and others in contact with persons with diabetes**

Circle the letter for the answer you believe to be correct. (Circle one only)

1. Diabetes diet is:

a. the food most people eat

b. a healthy diet for most people

c. too high in carbohydrates for most people

d. too high in protein for most people

2. Which contains most carbohydrate?

a. Oven-baked chicken

b. Hard, white cheese

c. Baked potato

d. Peanut butter

3. Which contains most fat?

a. Low fat milk

b. Orange juice

c. Sweetcorn

d. Honey

4. Which can be eaten ‘freely’ by persons with diabetes (do not increase blood sugar)?

a All unsweetened food

b. All diet foods

c. All food labelled “sugar-free”

d. All food that has less than 20 calories per serving

5. HbA1c (glycosylated haemoglobin) is a test which measures average blood sugar in the last:

a. day

b. week

c. 6-10 weeks

d. 6 months

6. Which is the best method for measuring blood glucose?

a. Urine testing

b. Blood testing

c. Both methods are equally good

7. What effect does unsweetened fruit juice have on blood glucose in a person with diabetes?

a. Lowers it

b. Raises it

c. Has no effect

8. Which should not be used to treat low blood glucose?

a. 3 hard candies

b. ½ cup orange juice (approx 100 ml)

c. 1 cup diet soft drink (approx 200 ml)

d. 1 cup of skimmed milk (approx 200 ml)

9. For a person in good diabetes control, what effect does physical activity have on blood glucose?

a. Lowers it

b. Raises it

c. Has no effect

10. In a person with diabetes infections is likely to cause:

a. higher blood glucose

b. lower blood glucose

c. no change in blood glucose

11. In a person with diabetes, the best way to take care of the feet is to:

a. check and wash them every day

b. massage them with alcohol each day

c. soak them for one hour each day

d. buy shoes a size larger than usual

12. For persons with diabetes eating food with a lower fat content decreases the risk for:

a. nerve disease

b. kidney disease

c. heart disease

d. eye disease

13. Numbness and tingling may be symptoms of:

a. kidney disease

b. nerve disease

c. eye disease

d. liver disease

14. Which of the following is usually not associated with diabetes:

a. vision problems

b. kidney problems

c. nerve problems

d. lung problems

15. Signs of ketoacidosis include:

a. shakiness

b. sweating

c. vomiting

d. low blood glucose

16. If a person with diabetes is sick with the flu, which of the following changes should be done?

a. Take less insulin

b. Drink less liquid

c. Eat more proteins

d. Test for glucose and ketones more often

17. If a person with diabetes have taken intermediate-acting insulin (NPH), he/she is most likely to have an insulin reaction (low blood glucose) in:

a. 1-3 hours

b. 6-12 hours

c. 12-15 hours

d. more than 15 hours

18. If you realise just before lunch time that a person with diabetes did not get insulin before breakfast. What should be done?

a. Skip lunch to lower the blood glucose

b. Give the insulin dose that he/she usually gets at breakfast

c. Give twice as much insulin as he/she usually gets at breakfast

d. Check blood glucose level to decide how much insulin to give

19. If a person with diabetes is getting an insulin reaction, he/she should:

a. work out

b. lie down and rest

c. drink some juice

d. take regular insulin

20. Low blood glucose may be caused by:

a. too much insulin

b. too little insulin

c. too much food

d. too little exercise

21. If a person with diabetes takes the morning insulin but skip breakfast, his/her blood glucose will usually:

a. go up

b. go down

c. remain stable

22. High blood glucose may be caused by:

a. not enough insulin

b. skipping meals

c. delaying snacks

d. high ketone level in urine

23. Which one of the following will most likely cause an insulin reaction (low blood glucose):

a. heavy exercise

b. infection

c. overeating

d. not taking insulin
